# Anode Catalysts in CO_2_ Electrolysis: Challenges and Untapped Opportunities

**DOI:** 10.1021/acscatal.1c04978

**Published:** 2022-01-04

**Authors:** Ádám Vass, Attila Kormányos, Zsófia Kószó, Balázs Endrődi, Csaba Janáky

**Affiliations:** Department of Physical Chemistry and Materials Science, Interdisciplinary Excellence Centre, University of Szeged, Aradi Square 1, Szeged H-6720, Hungary

**Keywords:** CO_2_ electrolysis, CCU, oxygen evolution reaction, electrocatalysis, pH effects

## Abstract

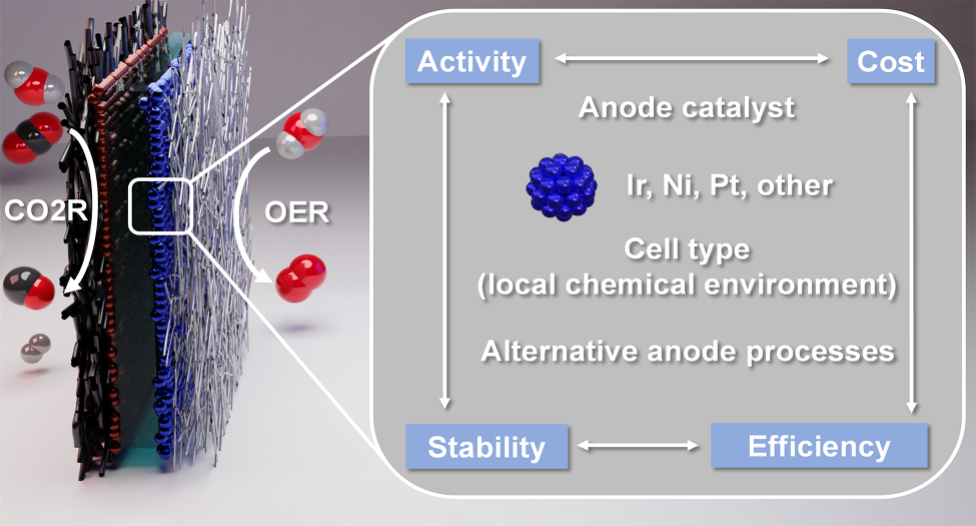

The field of electrochemical carbon dioxide reduction has developed rapidly during recent years. At the same time, the role of the anodic half-reaction has received considerably less attention. In this Perspective, we scrutinize the reports on the best-performing CO_2_ electrolyzer cells from the past 5 years, to shed light on the role of the anodic oxygen evolution catalyst. We analyze how different cell architectures provide different local chemical environments at the anode surface, which in turn determines the pool of applicable anode catalysts. We uncover the factors that led to either a strikingly high current density operation or an exceptionally long lifetime. On the basis of our analysis, we provide a set of criteria that have to be fulfilled by an anode catalyst to achieve high performance. Finally, we provide an outlook on using alternative anode reactions (alcohol oxidation is discussed as an example), resulting in high-value products and higher energy efficiency for the overall process.

## Introduction

Electrochemical conversion of carbon dioxide (CO2R) is an attractive way to simultaneously reduce atmospheric CO_2_ emissions and generate platform molecules that can be further processed to commodity/specialty chemicals.^1^ Although the first studies on CO2R date back several decades,^2^ the field has received broad and ever-growing attention only in the past 5–10 years.^3,4^ The driving force behind this increased interest is at least twofold. One is the awareness of society about the implications of the rising atmospheric CO_2_ concentration. This facilitates the decision-makers to support the research and development of technologies that could decrease the CO_2_ emissions while generating high-value products. The other motivator is the increasing amount of intermittently available electricity (originating from solar and wind energy), which brought the renaissance of electrochemical technologies. These offer a green and scalable alternative for energy storage and/or (in)organic synthesis.

A common feature in the most suitable electrolyzer cells of different structures is the continuously flowing fluid streams, removing the product(s) from the catalysts surface. A distinct attribute of such electrolyzer cells is that CO_2_ is fed in the gas phase to the cathode (and not as a CO_2_-saturated solution, which is typical in batch cells), where the catalyst is immobilized on a porous gas diffusion layer (GDL), together forming a gas diffusion electrode (GDE).^5^ This approach ensures that the diffusion length of the reactant is reduced by several orders of magnitude, leading to the intensification of the conversion process.^6^ The development of GDEs and electrolyzer cells enabled conversion of CO_2_ to methane, ethylene, formate, or carbon monoxide at a high reaction rate, approaching or even exceeding *j* = 1 A cm^–2^ (partial) current density.^7−12^ Industrially relevant reaction rates having already been achieved at acceptable energy efficiencies, more attention has been dedicated to the stability of such devices (i.e., operation for thousands of hours). Accordingly, processes hindering stability, such as electrode flooding or precipitate formation in the cathode GDE, are gradually getting better understood.^13−15^

Until recently, very little scrutiny has been devoted to the anode reaction and the anode electrode itself. Notably, during any electrochemical process, the oxidation and the reduction (the anodic and cathodic reactions) proceed at the exact same rate, and therefore the slower reaction will determine the total reaction rate. While this rate limitation is typically associated with the cathodic CO2R in aqueous solutions and in electrolyzer cells operating at low current densities, this might not be true at higher current densities and during long-term operation. As more and more studies report on the operation of CO_2_ electrolyzer cells at high current density, it has become necessary to take a closer look at the anode side of these cells.

**Figure 1:**
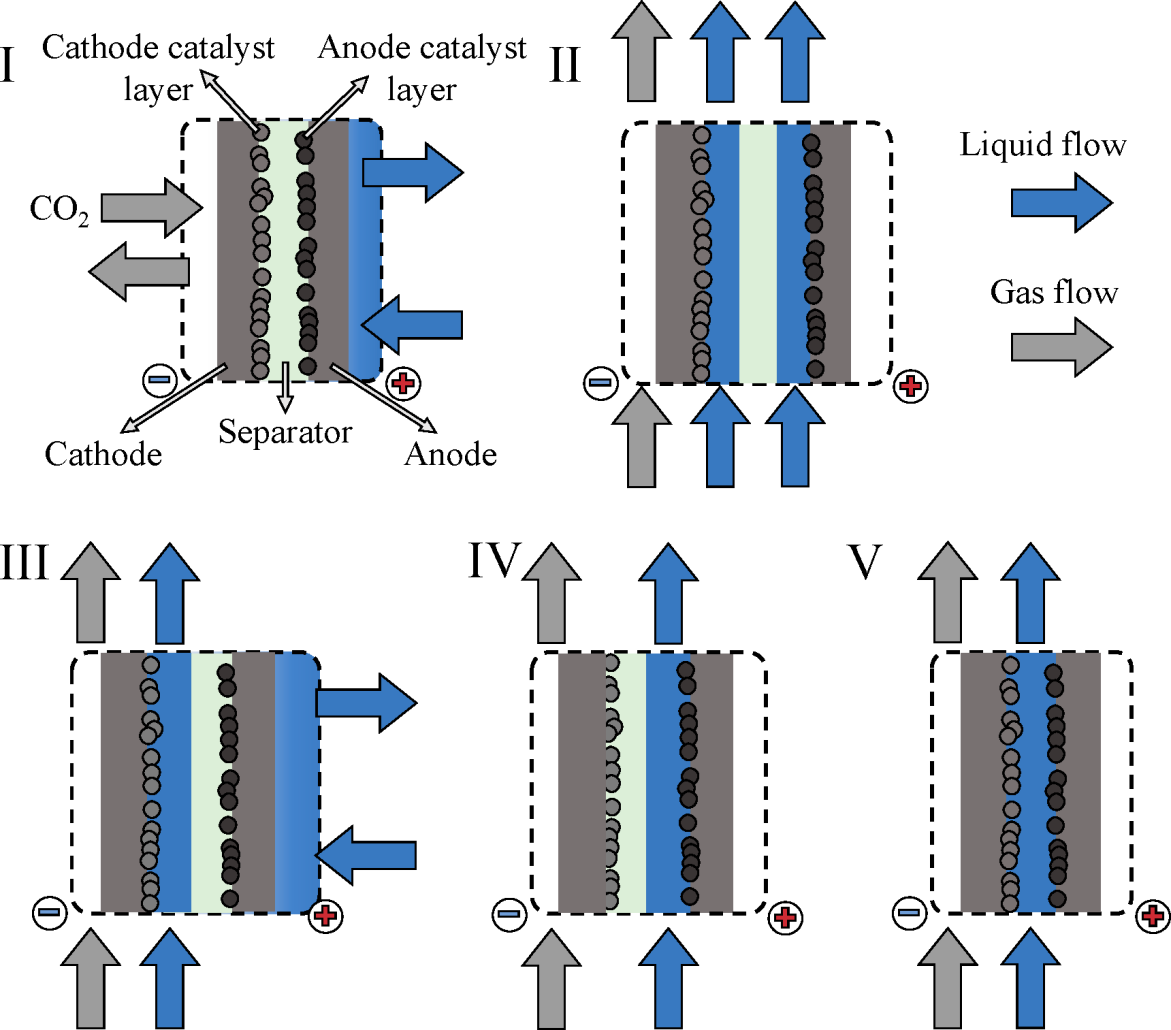
Schematics of the different cell structures employed in CO_2_ electrolysis.

The anodic process paired to CO_2_ electrolysis is typically the electrochemical oxidation of water to form oxygen (i.e., the electrochemical oxygen evolution reaction, OER). In most of the papers published on CO2R in continuous-flow electrolyzer cells, the anode catalyst is in contact with an (initially) alkaline electrolyte solution (anolyte; e.g., KOH). This provides conditions similar to the case of alkaline water electrolyzers. Recent studies, however, revealed that in the case of recirculating the anolyte it is neutralized during continuous operation.^16−18^ Furthermore, in electrolyzer cells where the catalysts are directly pressed to a separator (i.e., fuel cell type or zero-gap cells; see Figure 1), the surface chemistry might be significantly different from that in the electrolyte bulk, setting new requirements for the anode catalyst for stable operation. It is worth noting that these observations explain why Ir is a robust anode catalyst in CO_2_ electrolyzer cells, even though it is not stable in alkaline solutions.^18^

Performing OER as an anode process is preferred, as no mass transport issues are expected due to the presence of an ample amount of the reactant (>55 M reactant concentration) and the rapid removal of the O_2_ product. Moreover, water electrolysis is a well-studied and understood process, where a massive body of knowledge has been accumulated on the preferred anode catalysts, supports, binders, etc. On the other hand, a high positive thermodynamic potential is required for water oxidation (1.23 V), which is further increased by the overpotential, rooted in the kinetic hindrance of the OER on any known catalyst. Furthermore, the oxygen formed is of low value (∼30 €/t).^19,20^ To tackle these issues, increasing attention has been given to performing alternative anode reactions in conjunction with CO2R.^19,21,22^ As a specific example, oxidation of glycerol (e.g., to formate) occurs at several hundred millivolts lower potential in comparison to OER, which results in a lower electrolyzer cell voltage. This value-added approach increases the cost efficiency of the process, and therefore a rapid exploration of this field is expected.

In this Perspective, we do not aim to provide a comprehensive review on CO2R, as several thorough review articles are available on this matter.^23−27^ Our goal was to clarify what determines the chemical environment at the anode in continuous-flow CO_2_ electrolyzer cells and how this affects the overall performance. By analyzing and summarizing the results published during the past few years, we concluded what reaction conditions the anode catalyst must withstand during long-term operation in different electrolyzer cells. We paid special attention to those studies that reported exceptional performance from any aspect. We have also briefly reviewed studies on OER in near-neutral carbonate solutions, as these best resemble the CO2R process conditions during long-term operation. As an outlook, we highlight recent studies on coupling CO2R with alternative anode processes (especially alcohol oxidation).

## Electrolyzer Cell Types and Separators Used for CO_2_ Electrolysis

To identify potential anode catalysts for CO2R studies, it is essential to first understand and clarify what conditions develop in CO_2_ electrolyzer cells, under which the given catalyst must be stable and active for OER. Different electrolyzer cell types have been utilized for CO_2_ electrolysis during the past few decades (Figure 1).^3,28^ These differ in the number and properties of the applied fluids (i.e., gas and liquid streams) and the number (and structure) of the cell components. As detailed in the following, these seemingly minor variances lead to completely altered operation, setting very different requirements for the cell constituents (e.g., cell body, membrane, etc.) and for the catalysts.

On the basis of the above factors, at least five cell types can be identified. In zero-gap electrolyzer cells (Figure 1, cell type I) the electrodes are pressed to a separator by the current collectors, with the catalyst layers facing toward each other. A liquid electrolyte solution is fed to the anode, while CO_2_ gas is constantly supplied to the cathode. Hybrid electrolyzer cells (Figure 1, cell type II) resemble most closely the structure of regular H-cells. The electrodes are in contact with thin liquid layers (anolyte and catholyte), which are divided by a separator. The liquid electrolyte solutions are not static but are continuously flown by the electrodes, and gas-phase CO_2_ is fed to the cathode. When the separator is pressed directly to one of the electrodes (i.e., removing the anolyte or catholyte), two further variants of these hybrid cells can be derived (Figure 1, cell types III and IV). Finally, in microfluidic cells (Figure 1, cell type V) the electrodes are only separated by a continuously flowing liquid electrolyte solution (i.e., there is no solid separator). The laminar flow of the liquid is responsible for the removal of the formed products from the catalyst surfaces, hence avoiding the cross-talk of the electrode reactions.

Beyond the cell structure, the separator also affects the reaction conditions, by governing the local chemical environments at the cathode and anode sides. This effect is more pronounced when the separator is in direct contact with the catalyst layer(s). If there is a liquid layer between the membrane and the catalyst layer(s), the effect of the membrane is less direct, as the flowing electrolyte solution(s) serve as buffer layer(s), defining the local chemical environment (together with the electrode processes).

**Figure 2:**
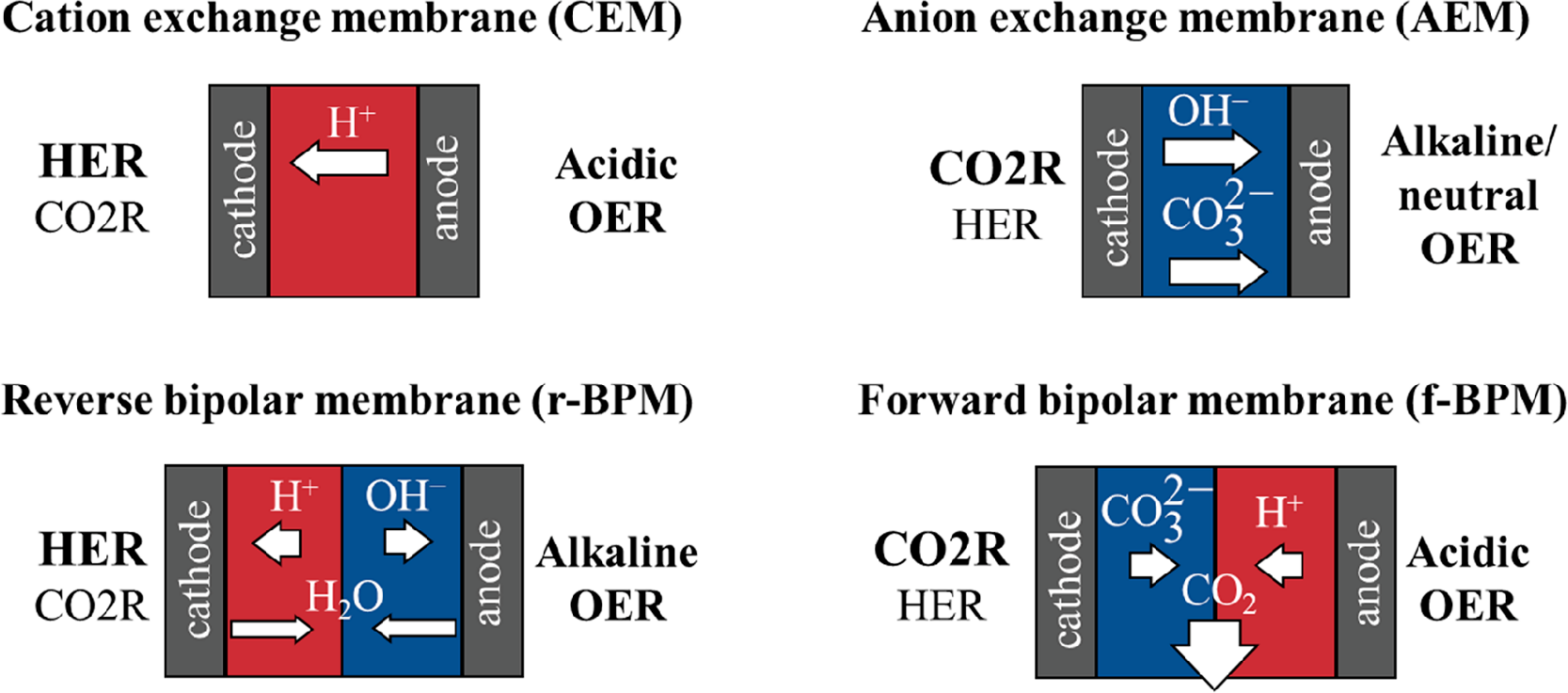
Schematics of the ion transport processes through different membranes during CO_2_ electrolysis in membrane-separated CO_2_ electrolyzer cells. The reactions favored due to the local chemical environment of the electrode, defined by the membrane, are highlighted in bold.

Inorganic diaphragms (e.g., ZrO_2_) might serve as separators,^29^ but ion exchange membranes are more frequently used due to their lower electrical resistance. Cation exchange membranes (CEMs), bipolar membranes (BPMs), and most frequently anion exchange membranes (AEMs) have been studied. As depicted in Figure 2, the membrane dictates the ion transport processes between the electrodes, and consequently, it determines the chemical environment at the membrane–catalyst interfaces (see also Table 1). In the case of CEMs, cations, most importantly H^+^ ions, migrate from the anode to the cathode. Using AEMs, the ion transport occurs in the opposite direction: anions, most importantly OH^–^, CO_3_^2–^, and HCO_3_^–^, migrate through the membrane from the cathode to the anode. BPMs consist of an AEM and a CEM, and therefore the ion conduction has two components.^30^ In the regular configuration (reverse BPM, r-BPM), the CEM is at the cathode side, while the AEM is at the anode side. In this case, water dissociates at the junction of the membranes (often facilitated by a thin catalyst layer),^31^ while OH^–^ ions move toward the anode and H^+^ ions to the cathode. When the order of the membranes is switched (forward BPM, f-BPM), the transport of H^+^ ions from the anode and of OH^–^, CO_3_^2–^, or HCO_3_^–^ ions from the cathode to the membrane junction maintain the ion conduction.

**Table 1:** Summary of Ion Transport Processes, the Conditions Emerging at the Membrane Surface, and the Main Obstacles during CO_2_ Electrolysis Using Different Ion Exchange Membranes

	surface pH		surface species			
	anode	cathode	anode	cathode	transporting ions	main obstacle(s)
CEM	acidic	acidic	H^+^, anolyte	H^+^, CO_2_, cations	H^+^, cations from anolyte	predominant cathodic HER
AEM	∼neutral	alkaline	CO_3_^2–^	OH^–^, CO_2_, HCO_3_^–^, CO_3_^2–^	OH^–^, HCO_3_^–^, CO_3_^2–^, (cations from anolyte)	CO_2_ crossover
r-BPM	alkaline	acidic	OH^–^, anolyte	H^+^, CO_2_	H^+^ and OH^–^	Predominant cathodic HER
f-BPM	acidic	alkaline	H^+^, anolyte	OH^–^, CO_2_, HCO_3_^–^, CO_3_^2–^	OH^–^, HCO_3_^–^, CO_3_^2–^ and H^+^, cations from anolyte	CO_2_ formation between the membranes

The membrane choice is vital for multiple reasons. The most trivial is that the ion conduction—which directly affects the cell voltage—depends on the mobility of the charge carrier species and hence on the membrane type. The high mobility of H^+^ ions and the well-developed, thin yet robust CEMs together offer the lowest cell resistance. Using CEMs, however, leads to an acidic surface pH at the cathode side of the membrane due to the H^+^ conduction. The high H^+^ flux can be avoided by applying concentrated anolytes: cations from the anolyte (e.g., K^+^) maintain the ion conduction between the electrodes.^7,8,32^ However, this results in a high local cation concentration at the cathode, where a carbonate precipitate might form, fading the performance of the electrolyzer cell.^13^ In cells where a catholyte flows between the cathode and the membrane, the high H^+^ concentration at the cathode side of the membrane has only a minor influence on the cathode surface pH, as that is mostly dictated by the liquid electrolyte. On the other hand, if the membrane is directly pressed to the cathode (Figure 1, cell types I and IV), the high surface concentration of H^+^ leads to a favored hydrogen evolution reaction (HER).^33^ For AEMs, the ion conduction is maintained mainly by CO_3_^2–^ (and OH^–^) ions under process conditions.^12,13,34^ This leads to an alkaline pH at the cathode side of the membrane (which is favorable for CO2R), while a high carbonate ion flux reaches the anode side of the membrane. If a liquid layer flows by the anode (cell types II and IV), this high carbonate concentration is diluted, and therefore it might not affect the anode catalyst significantly. When the anode is pressed to the membrane (cell types I and III), however, the carbonate ion flux can detrimentally affect the stability of the anode catalyst. Another necessary consequence of the carbonate transport was revealed during long-term experiments with recirculated anolyte: the pH of the anolyte changes to near-neutral, even if a highly alkaline solution was applied at the beginning of the experiments.^17,18^ This sets new requirements for the anode catalyst that must therefore be stable and active in OER at near-neutral pH and, in some cases, even in concentrated carbonate solution.

BPMs comprise a group of interesting, but less frequently studied, separators in CO2R studies. In regular operation (r-BPM), acidic and alkaline environments develop at the cathode and anode sides, respectively. This again leads to increased HER selectivity at the cathode, if there is no buffer layer included between the membrane and the catalyst layer (cell types I and IV). A further drawback of using r-BPMs is the increased cell voltage, rooted in the additional membrane–membrane interface (and possible water dissociation catalyst) and in the voltage needed to facilitate water dissociation at the membrane–membrane interface.^31^ In the case of f-BPMs, the cathode is alkaline, while the anode is acidic. In this configuration, the ion transport processes are both toward the junction of the membranes, where neutralization occurs. Depending on the charge carrier ions (CO_3_^2–^/HCO_3_^–^/OH^–^ from the cathode, H^+^ or metal cations from the anode), water, metal carbonates, or CO_2_ forms at the membrane–membrane junction. Water and metal carbonates can lead to electrode flooding and resistance increase, respectively.^30^ In zero-gap cells (type I), the most probable scenario is carbonate conduction from the cathode and proton conduction from the anode (due to the high mobility of H^+^ in the typically used CEMs). In this case, CO_2_ is liberated from the reaction of H^+^ and CO_3_^2–^ ions. This gas formation leads to the physical separation and eventual mechanical failure of the membranes.^35^ Note that this occurs in all types of cells, even if liquid electrolytes are in contact with the BPM, and it is therefore not trivial to operate an f-BPM-separated CO_2_ electrolyzer cell.

## Selection Criteria for Studies to Be Included in Our Analysis on OER Catalysts

**Scheme 1:**
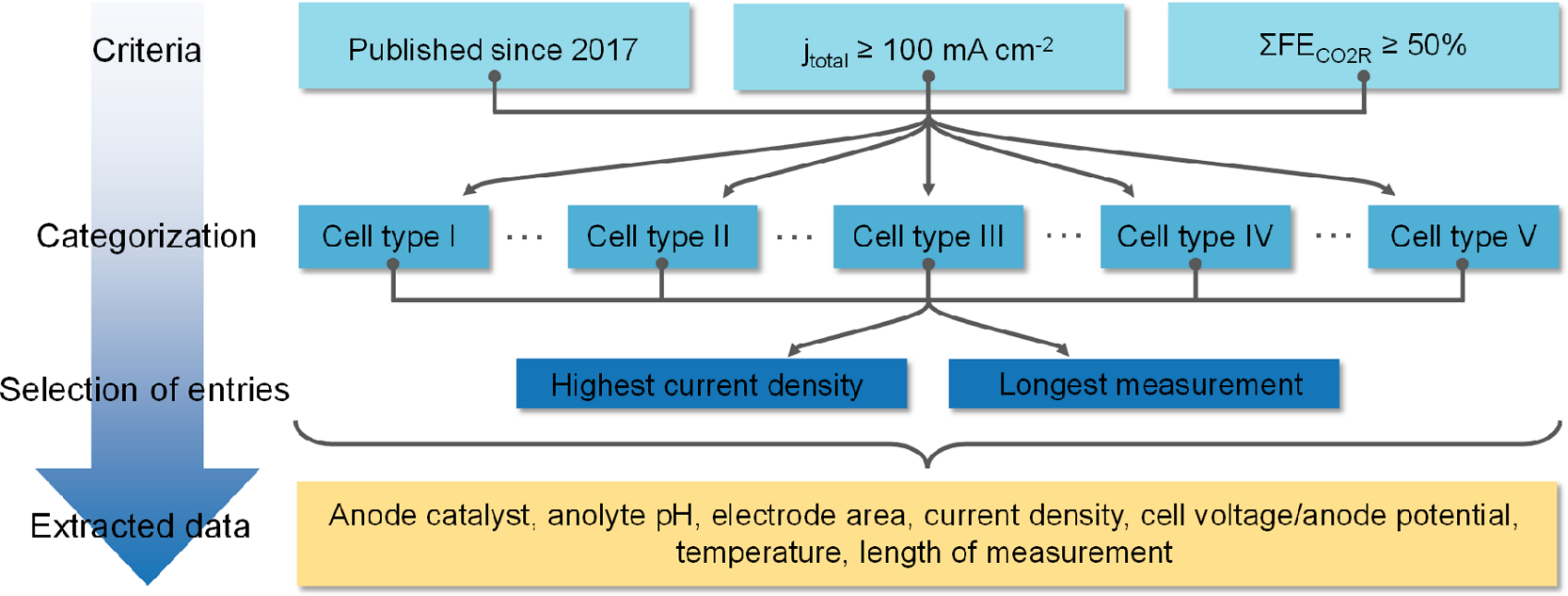
Visual Summary of How the Analyzed Entries Were Selected from the Literature for this Perspective

In this Perspective, we limited our scope to the past 5 years. We note that, even in this relatively short period, an exponential increase in the publication rate was observed. To avoid losing focus, we defined a set of criteria that should be simultaneously fulfilled to be included in our analysis (Scheme 1). First, the reported current density should reach at least 100 mA cm^–2^, and at least 50% of this must be consumed by the formation of CO2R products (this also means that the partial Faradaic efficiency of each product must be accurately reported). If these criteria were fulfilled for multiple measurements in a given paper, two entries were created: one containing the highest achieved current density (regardless of the duration of the experiment) and another with the current density applied/measured during the longest reported measurement. If more than one cell type was investigated in the same paper, the number of entries was multiplied by the cell types.^10,36−45^

Unfortunately, numerous parameters (e.g., the length of the given experiment, pH over the course of the experiments, etc.) are poorly reported in a considerable number of studies. In some extreme cases, even such crucial parameters as the size of the electrolyzer cell, the anode catalyst employed, and/or the applied voltage/current density are missing. If the length of the given experiment was not provided, 10 min of measurement time was estimated (enough for a coupled gas chromatography measurement with a short program). Similarly, if the pH of the anolyte was not monitored throughout the experiments, it was estimated from the initial conditions (electrolyte composition). Our analysis is based on a total of 121 articles in which the reported measurements met the above requirements, representing a total of 209 entries. The number of entries used to create each figure may differ from the total quantity due to the unknown parameters detailed above.

## OER Catalysts Studied in CO_2_ Electrolyzer Cells so Far

**Figure 3:**
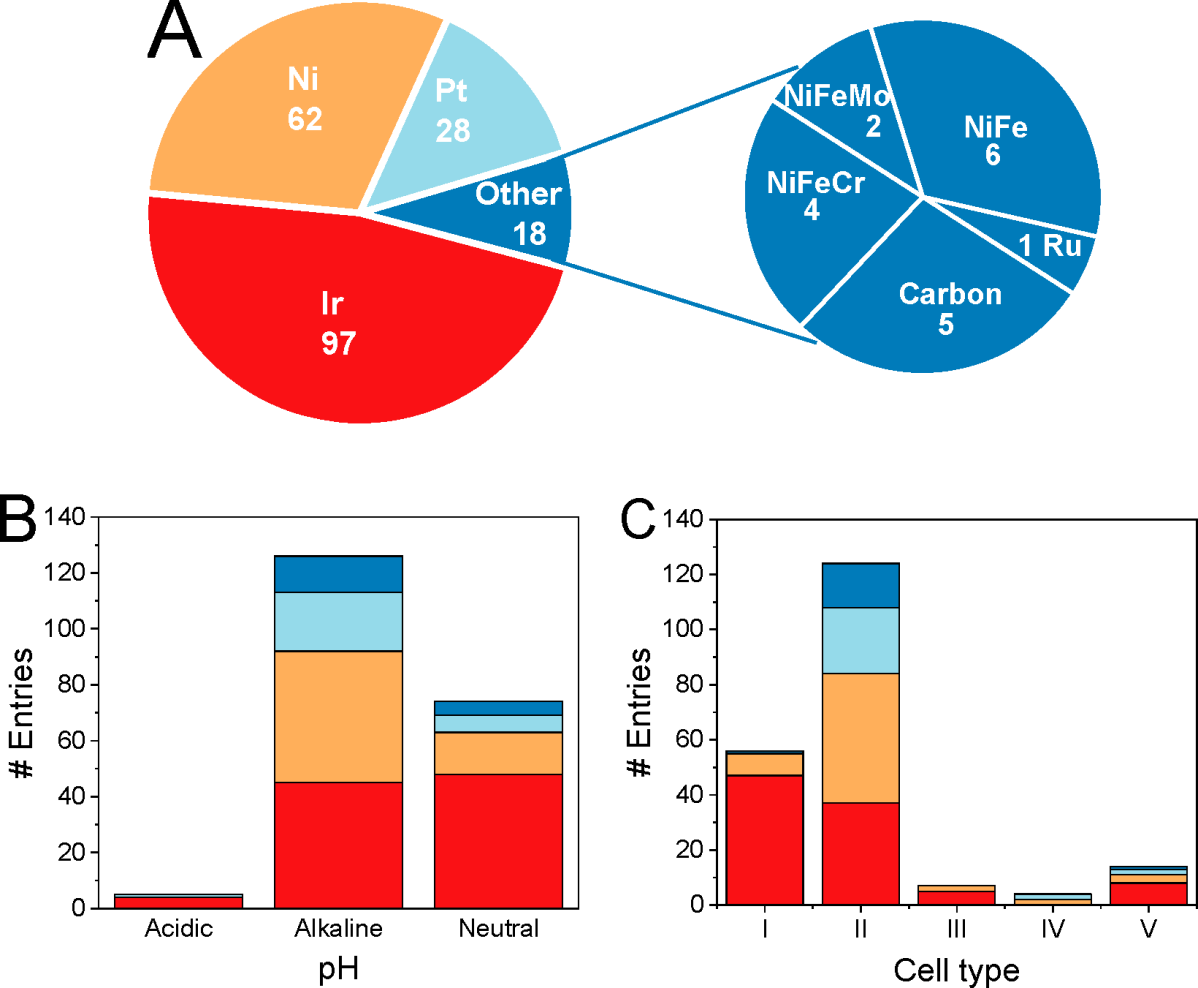
(A) Pie chart showing how often a given anode catalyst is used in the articles referenced in this Perspective. The numbers reflect the number of entries created from the inspected literature references. Diagrams showing how frequently a given anode catalyst was studied (B) under acidic, alkaline, and neutral pH and (C) in different CO_2_ electrolyzer cell types. The colors consistently indicate the different catalysts. Data points were gathered from refs (7−13, 16−18, 29, 32, 34, and 36−140).

In almost half of the cases (47%), Ir was employed as the anode catalyst,^10,12,13,16,18,29,32,34,36−87^ Another 30% and 14% account for Ni^7−10,17,36,39,41−44,88−117^ and Pt,^32,114,118−133^ respectively, while only 9% is related to other metals, metal alloys, or carbon^11,45,60,117,134−140^ (Figure 3A). The dominance of Ir and Ni is rooted in the fact that these are the generally used catalysts in acidic and alkaline water electrolyzers, respectively. The frequent use of Pt is unexpected, considering that Pt is not among the most active OER catalysts in neither acidic nor alkaline medium.^141^ We assume that choosing Pt might be reasoned by its acceptable stability during laboratory experiments and by the relevant experience of researchers with this catalyst. The alloys are all nickel alloys; however, we considered it important to distinguish between metallic Ni and its alloys.

CO2R studies are mostly performed in alkaline media (Figure 3B). The role of alkaline electrolytes at the anode is at least twofold. One is to ensure high conductivity and reduce cell resistance, resulting in an overall lower cell voltage. Additionally, part of the cations present in the anolyte can cross over to the cathode side under the operating conditions. As mentioned above, a high alkali cation concentration results in an excessive precipitate formation at the cathode side, which is detrimental for stable operation.^64,142^ On the other hand, in cells operating without liquid catholyte (cell types I and IV) the slow crossing of cations through the AEM during electrolysis could boost cell operation, and the presence of a small amount of cations at the cathode surface is necessary to achieve a high CO2R rate.^13,143^

Ni or Ni-based electrocatalysts are dominantly used under alkaline conditions due to their remarkable activity and stability. Interestingly, Ir is the second most frequently applied electrocatalyst, despite its known slow dissolution in highly alkaline solutions.^18^ This might be because of the short duration of the experiments or because of the gradual decrease of the anolyte pH to a near-neutral value during continuous operation, as detailed above. About one-third of the studies employed near-neutral electrolytes at the anode. The trend concerning Ir and Ni is reversed here, in favor of Ir. This is not surprising on the basis of the dissolution of Ni in neutral solutions, while Ir is stable (and active) during neutral pH OER.^18,144^ Finally, only a handful (five) of the entries can be found under acidic conditions.^10,32,61^

It is clear from Figure 3C that only three of the aforementioned five cell types are frequently used: microfluidic cells operating without membrane separation (V), zero-gap cells (I), and hybrid cells (II), the last two together accounting for about 90% of all studies. At first sight, the dominance of hybrid electrolyzer cells is surprising, as their precise operation requires a complex test environment (two liquid pumps, mass flow controller, pressure controllers, etc.). Their frequent use, however, is understandable, as their structure is the most similar to that of H-cells, typically employed in laboratory experiments (“continuous-flow H-cells”). Additionally, these cells allow controlling the local pH at the anode and cathode separately, therefore providing optimal conditions for each process. Zero-gap CO_2_ electrolyzer cells build on the knowledge gathered on PEM and AEM water electrolysis, which also explains their prevalence. Their simple structure and operation offer a relatively easy path for industrial implementation. Finally, microfluidic cells are simple and cost-effective platforms for testing new catalysts in CO2R, which makes these ideal for rapid screening experiments.

**Figure 4:**
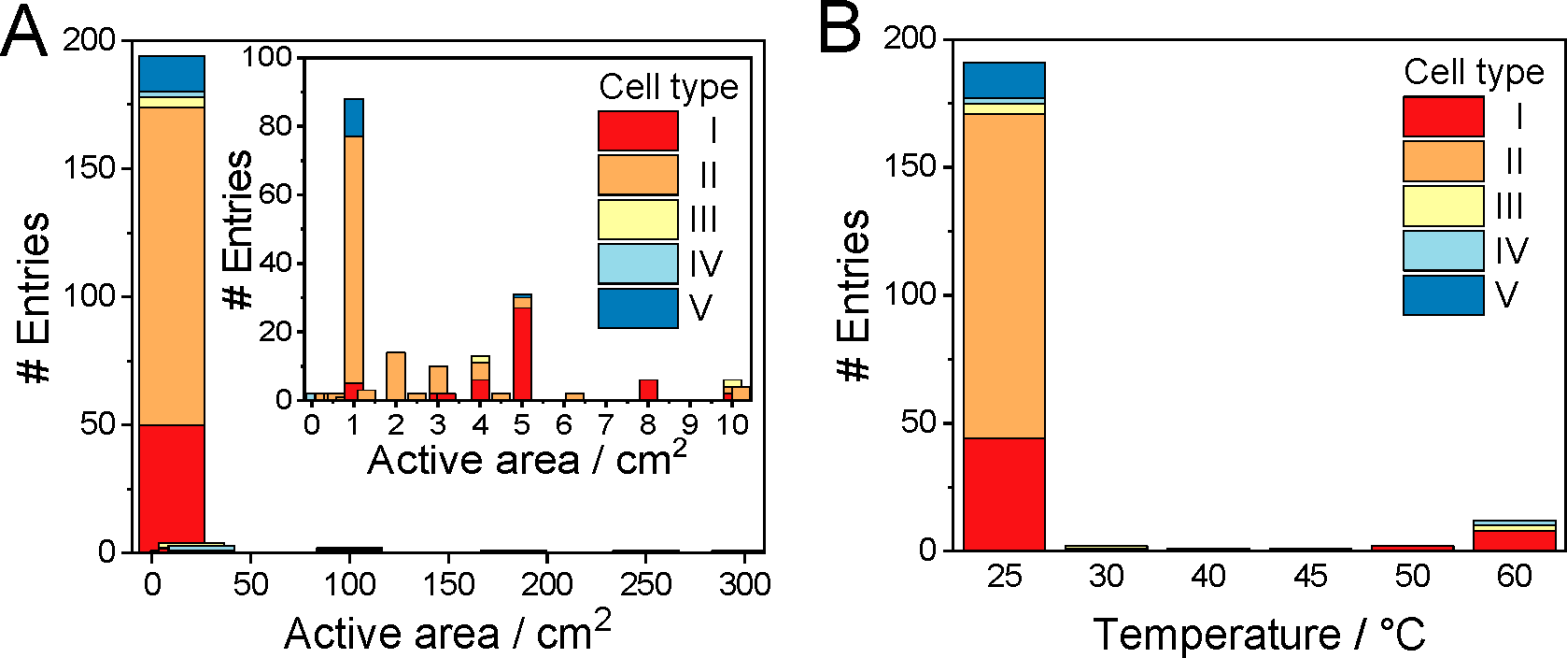
Diagrams showing (A) the geometric area distribution of the electrolyzer cells used (data points were gathered from refs (7−13, 16−18, 29, 32, 34, 36−140, 145, and 146)) and (B) the applied cell types as a function of the applied reaction temperature (data points were gathered from refs (7−13, 16−18, 29, 32, 34, 36−140, and 145−147)).

The vast majority (∼85%) of the studies were performed in electrolyzer cells with a geometric surface area smaller than 5 cm^2^ (Figure 4A). Even more importantly, in almost half of the cases, the electrolyzer cell size used was 1 cm^2^ or smaller. While these studies report important and valuable findings, it is worth mentioning that, in such a small size, the edge effects might seriously distort the results. Furthermore, bubble management, heat management, and reactant transport might be notably different in larger electrolyzer cells; therefore, it is not trivial to transfer this knowledge to industrially relevant conditions (i.e., large electrolyzer cells and stacks).

The geometric surface area of the electrodes in the electrolyzers reached or approached 100 cm^2^ in the case of only five entries, all of them operated with Ir catalyst in membrane-separated cells. This suggests that upscaling single electrolyte solution separated microfluidic electrolyzers (type V) might not be a viable option. The reason behind this could be the cross-talk of the electrode processes that becomes more intense in larger electrolyzer cells, where a longer residence time of the electrolyte solution assures more time for product transport to the other electrode.

Continuous-flow CO2R measurements are typically performed at room temperature (Figure 4B). This is indeed surprising, especially since the sluggish kinetics of OER is typically boosted in water electrolyzers by increasing the reaction temperature. It is noteworthy that Ir-based PEM water electrolyzers operate in the 60–80 °C temperature range, with an expected lifetime of 60000–80000 h.^148^ For CO_2_ electrolyzer cells operating at room temperature, we note that the inner temperature of the cells is very seldom reported; the values—at least to our understanding—refer to the initial temperature of the anolyte/catholyte (i.e., ambient temperature). Inside the cell, however, the temperature increases due to the Joule heating effect. The electrolyte solutions are typically pumped at a very low rate in cell types II and V, and therefore the inner temperature of these cells during operation could be significantly higher than the ambient temperature. Zero-gap cells (type I) are more often operated at elevated temperatures. The anolyte recirculation rate is typically 10–100 times higher than that in microfluidic/liquid flow cells, allowing better temperature control.

**Figure 5:**
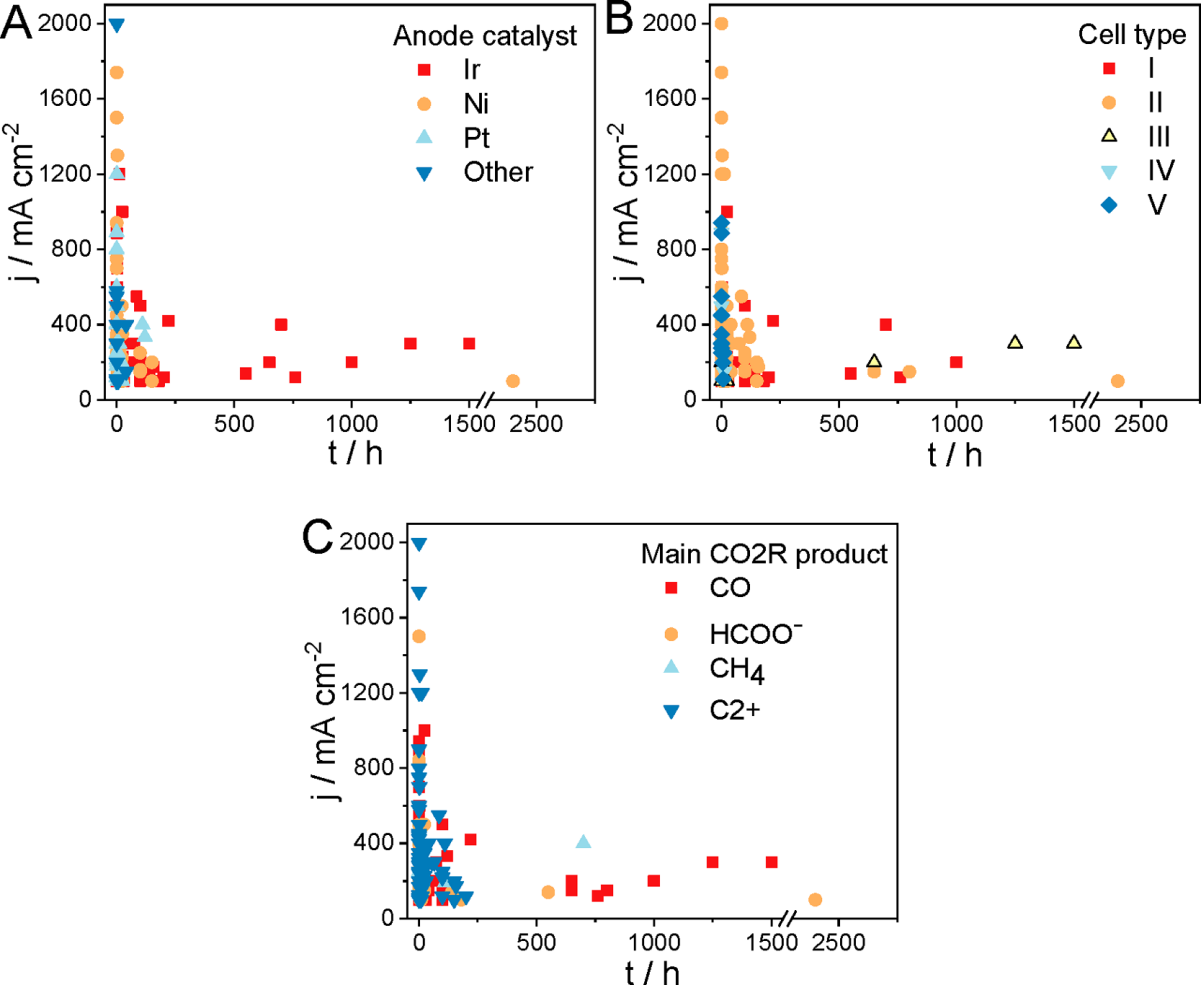
Current density as a function of the length of the experiment, marking (A) the different anode catalysts (data points were gathered from refs (7−13, 16−18, 29, 32, 34, and 36−140)), (B) the different electrolyzer cell structures, and (C) the formed main CO2R products (data points for (B) and (C) were gathered from refs (7−13, 16−18, 29, 32, 34, 36−140, and 145−147)). C2+ corresponds to any multicarbon product where the number of C atoms is equal to or higher than two.

Analyzing the highest reported CO2R current densities, values over *j* = 1 A cm^–2^ were only reported in cell type II^7−11,32^ (with one exception performed in cell type I^12^) and only in very short measurements. The reason behind this might be the high cation concentration at the cathode surface ensured by the concentrated catholyte solution (Figure 5A).^7,8,32^ The electrolyte flow also provides efficient product removal from the catalyst surface. At the beginning of these experiments, the existence of a real triple-phase boundary is envisioned (note the problem of short reaction times again!). During longer measurements, electrowetting and other structural changes might occur in the GDE, leading to higher water content and the formation of a double phase boundary (solid/liquid) in the catalyst layer. These very high initial current densities are mostly transients and should therefore be handled with caution in aiming for industrial application. It is a typical trend to report transient high current densities and perform longer measurements at much lower current densities. In our opinion, this is acceptable until the difference between the highest presented current density and that used for stability demonstration is not too large (i.e., <50%). Showing a strikingly large current density but performing long measurements at 10–20% of this value is, however, very misleading. Interestingly, Ni (or a Ni alloy) is the preferred choice of anode catalyst in the highest current density reports.^7−11^ Since a highly alkaline media at the anode is guaranteed by the anolyte in cell type II, which remains unchanged during short measurements, Ni is a stable anode catalyst.

The current density values decay rapidly with the length of the experiments (Figure 5), which suggests rapid cell failure at high reaction rates. This is typically attributed to unfavorable changes in the membrane or in the cathode GDE (e.g., flooding, precipitate formation, etc.). At the same time, changes in the anode catalyst morphology, composition, etc., are often overlooked and not studied. Less than 15% of the publications report on measurements longer than 100 h, and just a handful of reports can be spotted with measurements longer than 200 h. With one exception,^41^ the longest measurements (>200 h) were performed using an Ir anode catalyst.^13,29,43,52,76,79,80,85^ This further proves that Ir is stable under process conditions (i.e., the anolyte pH decreases to a near-neutral value, hence avoiding the dissolution of Ir).^18^ For the exceptionally long measurement with Ni anode catalyst,^41^ it must be emphasized that (i) the measurement was performed in a hybrid electrolyzer cell, which allows control of the local chemical environment at the electrodes separately and (ii) the anolyte was periodically regenerated and hence its pH never decreased below pH 11. This way, the alkaline environment was guaranteed at the anode during the whole measurement, ensuring that the Ni catalyst remained stable. Although this might be a viable approach (depending on the operational cost), it needs continuous monitoring and controlling of the anolyte composition and an extensive use of alkaline anolyte.

For the cell type, no apparent trend can be seen in the length of the experiments (Figure 5B). Measurements longer than 100 h are shown in cell types I–III in an almost equal number of studies. This again signals that cell types I and II are preferred in CO2R studies, but the long-term studies in cell type III call attention to the applicability of these devices (taking into account, of course, the high cell voltage resulting from the cell construction).^76^ What limits the lifetime of these electrolyzer cells is not detailed in most cases. Post-mortem analysis of the cell elements^142^ (including all the MEA components and the cell hardware) is mostly lacking; therefore, it is difficult to identify the most important failure mechanisms. The latter is not very surprising, as the field is in its infancy; only a small number of the publications report on longer experiments, where such fading mechanisms might appear.

The degradation of the anode catalyst is an important but often neglected aspect of stability studies. As was mentioned above, a possible anode fading mechanism is catalyst dissolution, caused by the governing local chemical environment. The importance of other degradation mechanisms, however, can be envisioned similarly to water electrolyzer cells.^149^ Similarly to the case of the cathode catalysts,^142^ these include the following.

- Physical/chemical degradation of the catalyst layer, which includes the dissolution, detachment, and delamination of the catalyst particles and also the chemical/physical corrosion of the catalyst binder (e.g., PTFE or ion exchange ionomer).
- Particle aggregation, which leads to the decrease of the electrochemically active surface area and to the blockage of the gas channels.
- Catalyst poisoning (i.e., by cell component dissolution, by cathodically formed products, etc.), leading to decreased catalytic activity and hence an increased anode potential and cell voltage.
- Degradation/passivation of the electrode support (i.e., oxide layer formation on a porous Ti electrode, or overoxidation of carbon-based electrodes), leading to increased cell resistance. Furthermore, because of the passivation of parts of the anode, higher local currents are driven through some parts of the catalyst layer. This high local current density (hot spot) can accelerate the catalyst (and membrane) degradation.

In terms of the reduction products, CO and HCOO^–^ were the main products during the longest experiments, both formed via the transfer of two electrons (Figure 5C). There is one outlier entry, reporting a stable CH_4_ production (i.e., eight-electron process) in a zero-gap cell for 700 h.^85^ The highest current densities were reported for systems where C2+ formation was the main CO2R pathway. High current densities, however, go hand in hand with short measurement times, highlighting that the partial crossover of the liquid products formed to the anode side can influence the operation of the electrolyzer. This is inevitable for all currently used AEMs, where negatively charged products (e.g., acetate and formate) can transport through the membrane via electromigration, while neutral liquid products (such as alcohols) can cross via diffusion and electroosmotic drag.^150^ In extreme cases it can lead to the loss of 30–40% of the formed products.^53^ As a next step, the migrated liquid products can be partially or fully oxidized at the anode. Several mitigation strategies are already in development, including tailoring the water uptake of the AEM (could help with the retention of neutral products), along with introducing product-blocking functional groups on the membrane surface and applying differential pressure between the anode and cathode in a zero-gap cell configuration.^150,151^

As a final remark on the reported results, we mention that the anode potential is provided in only a small fraction of the studies, as the focus is typically on the development of CO2R cathode catalysts. It is therefore not yet possible to compare the intrinsic activity of the different anode catalysts among the various studies.

## Alternative Anode Catalysts for the OER

As detailed above, for the long-term operation of CO_2_ electrolyzer cells either the anolyte must be continuously refreshed to maintain a highly alkaline pH or an anode catalyst should be used that is stable in OER at near-neutral pH. For the latter, Ir is stable; however, economic reasons urge the exploration of alternative OER catalysts. Water oxidation at near-neutral pH would also allow the use of cheap structural materials for constructing electrolyzer cells. The exploration of near-neutral pH water oxidation catalysts has therefore been long pursued.^152−156^ The knowledge and experience gathered in these related fields might be used as a background in searching for new anode catalysts for CO2R studies. Importantly, the catalytic activity of the catalyst candidates must be tested in relatively high concentration carbonate/bicarbonate buffer solutions.

The majority of the studies on near-neutral pH OER were performed in phosphate and borate buffer solutions. Only a few dozen papers were published on OER in carbonate buffers. These include studies with Co-, Fe-, Ir-, and Ni-based materials.^152−156^ A common point in these studies is the interaction between the catalyst and the carbonate/bicarbonate ions. This way, the active material on the catalyst surface is generated *in situ* during the OER experiment. Recent studies demonstrated that carbonate ions could participate in the water oxidation reaction, leading to the formation of different radicals and also increasing the probability of peroxide formation.^157,158^ These considerations set important requirements for a potential anode catalyst in CO_2_ electrolyzer cells: namely, it must either remain unchanged during the reaction or the compound forming in its reaction with carbonate ions must be active for OER and it must be insoluble in the applied aqueous solution to avoid extensive catalyst loss.

## Value-Added Anode Processes: Alternatives to the OER

**Figure 6:**
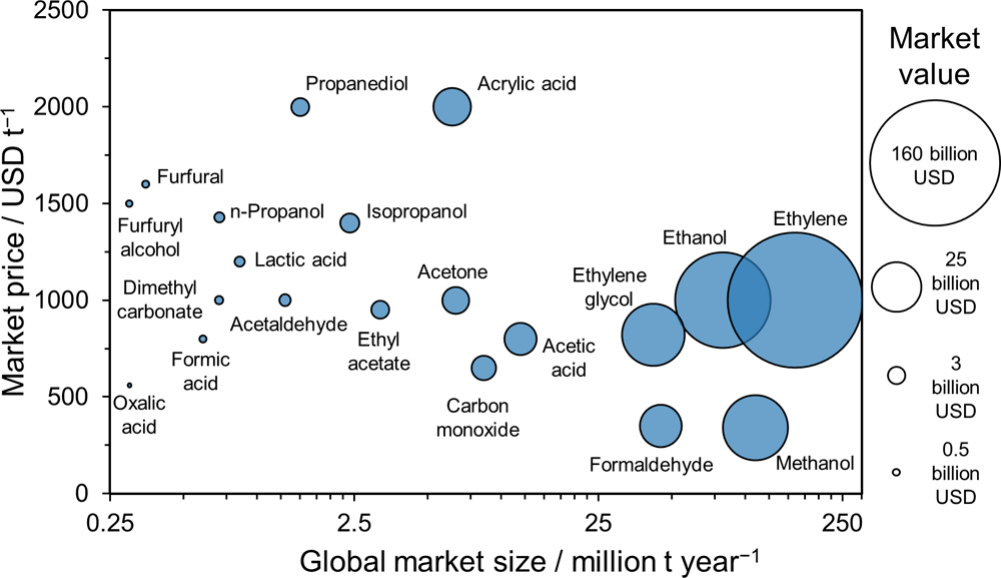
Market potential of chemicals possibly produced by coupling CO2R with organic oxidation reactions (our compilation).

From a purely thermodynamic perspective, it was found that more than 90% of the overall energy required to operate a CO_2_ electrolyzer cell is associated with the anodic OER.^22^ This issue could be circumvented by coupling CO2R with alternative anode processes, occurring at less positive potentials. This would result in lower cell voltage along with the possible generation of high-value products at the anode. Such alternative processes could be the electrocatalytic oxidation of chloride ions, aliphatic and aromatic alcohols, amines, urea, hydrazine, and several biomass-derived compounds (e.g., 5-(hydroxymethyl)furfural (HMF), sorbitol, etc.).^159−164^ In addition, coupling value-added anode processes with CO2R maximizes the potential of utilizing waste carbon sources to generate valuable products. These circular processes will certainly play a key role in putting the chemical industry on a more sustainable path. Some products and their market potential are summarized in Figure 6, on the basis of our own literature and market survey (note that even the smallest dots represent USD ∼0.5 billion market value).

A crucial criterion, which is often neglected in publications, is to ensure the overall (“from CO_2_ source to the final products”) carbon-neutral/-negative operation of the CO_2_ electrolyzer (i.e., we are not just using a sacrificial electron donor and generating CO_2_). To realize this goal, several factors, such as the targeted products (e.g., the comparative scale of production of CO2R and small organic molecule oxidation products), the source/purity of the substance to be oxidized (e.g., byproduct/waste stream), the overpotential, and the selectivity of the anode reaction have to be *simultaneously* considered. In the existing literature, electrocatalytic alcohol oxidation (AOR) is the anode process most commonly paired with CO2R; therefore, the progress of this new research direction is demonstrated for that example.

## Alcohol Electrooxidation to High-Value Products Paired with CO_2_ Electrolysis

While there are many examples that perform HER in parallel with electrocatalytic AOR, only a handful of reports can be found on its pairing with CO2R.^22,162,165−171^ Even more importantly, only three of these were performed in continuous-flow cells.^22,165,166^ Type II cells were used in all studies with either strongly alkaline (2 M KOH) or near-neutral electrolytes (0.5 M KHCO_3_). While driving AOR at the anode notably decreased cell voltages in all cases, the current densities achieved remained relatively low (below 100 mA cm^–2^) and long-term stability was not demonstrated (the longest measurement was performed for 24 h, but current densities were below 10 mA cm^–2^).^166^ Glycerol,^22,166^ glucose,^22,166^ methane,^22,166^ and 1,2-propanediol^165^ oxidations were also tested as anode processes. Out of these, glycerol oxidation resulted in the lowest cell voltage (1.5 V^22^ and 1.55 V^166^ when Pt/C or Ni_0.9_Au_0.1_ was used as the anode catalyst, respectively).

The rest of the cited studies were carried out in stagnant electrolytes, employing a membrane-separated H-cell configuration. The list of both the studied substances and electrocatalysts are more diverse here: in addition to glycerol,^167^ HMF,^167,170^ methanol,^168^ ethanol,^169^ isopropanol,^171^ 1-phenylethanol,^171^ 4-methoxybenzyl alcohol,^171^ and benzyl alcohol^162^ were tested as potential oxidizable substance candidates (sometimes called fuels). Both heterogeneous (noble metals such as Pt and Pd, metal oxides such as CuO nanosheets and NiO nanoparticles, and a redox mediator (STEMPO)) and dissolved^162^ electrocatalysts were considered. Similarly to the measurements in flow cells, substituting OER by AOR at the anode led to a decrease in the cell voltages. However, current densities fall behind those typically applied/measured in the standard CO2R/OER scenario (a maximum of 10–15 mA cm^–2^). As the AOR can follow several pathways (e.g., glycerol oxidation can proceed toward either glyceraldehyde or dihydroxyacetone)^163^ the selectivity has a key importance. In this vein, the effect of the cell parameters (e.g., cell voltage, temperature, and current density) on product selectivity should be analyzed further.

Overall, all preceding research on paired CO2R/AOR has been based on model studies, employing commercially available electrocatalysts or redox mediators that were proven to be active toward AOR in strongly alkaline solutions. Therefore, there is a huge room for improvement in terms of the applied electrocatalyst–substance pairs and the optimal operating conditions. The main challenges to be solved in the foreseeable future can be summarized as follows:

### Electrocatalyst

The ideal electrocatalyst should show high activity and selectivity toward the desired AOR at neutral pH (due to the neutralization effect discussed earlier). In addition, the given electrocatalyst should bear with excellent CO_3_^2–^ tolerance. Moreover, intermediates/products formed during the AOR should not adsorb irreversibly on the catalyst surface, leading to a gradual decrease of the activity.

### Oxidizable Substance (Fuel)

Little to no care was given in all the existing literature to the selection of the anode reaction by considering the overall carbon balance, except for one report.^22^ The only goal was to decrease the cell voltage in parallel with generating value-added products. In the long run, however, the anode reaction should be selected in a way that ensures the CO_2_-neutral or -negative operation.

### Operating Conditions

To move toward industrial applications, promising electrocatalyst candidates should be tested in continuous-flow cells. An optimum result should be found in terms of the electrolyte and substance concentrations. Moreover, the effect of temperature and elevated pressure on the selectivity of the AOR has to be scrutinized. The crossover of both the oxidizable substance and the products formed at the cathode side has to be considered, as they might cause the flooding of the cathode GDE. This means that novel membranes have to be developed bearing an improved substrate/product retention.

## Summary

We have summarized recent reports on CO_2_ electrolyzer cells operating at high current density from the last 5 years, with the focus on the anodic half-reaction. We have analyzed these studies from the perspective of the applied anode catalyst, electrolyzer cell (fluid inlets, structure, size), and operational parameters (pH, ion transport, temperature). We concluded that each cell type allows a different level of control during operation over the anode and cathode surface pH values, which are in some cases entirely determined by the applied cell type and the separator (and not the employed electrolyte). This fact limits the pool of applicable structural elements and catalysts. We have shown that the neutralization of the recirculated anolyte implies that the anode catalysts must be stable under near-neutral OER conditions. This explained the fact that Ir was stable under such conditions, while Ni was applicable in cells where the alkaline conditions at the anode were continuously ensured. Possible Ir replacements are catalysts that possess high OER activity and stability under near-neutral conditions along while they tolerate a high CO_3_^2–^ ion concentration. We have also uncovered that most studies reporting exceptionally high current density were carried out for very short timeframes, where no steady state can be expected either at the cathode or at the anode. Finally, we briefly outlined the opportunities and major challenges for coupling organic oxidation reactions to CO2R, showing that it is equally a catalyst and membrane challenge.
